# Serum Autoantibody Measurement for the Detection of Hepatocellular Carcinoma

**DOI:** 10.1371/journal.pone.0103867

**Published:** 2014-08-05

**Authors:** Catrin H. Middleton, William Irving, John F. R. Robertson, Andrea Murray, Celine B. Parsy-Kowalska, Isabel K. Macdonald, Jane McElveen, Jared Allen, Graham F. Healey, Brian J. Thomson, Stephen J. Ryder, Stefan Holdenrieder, Caroline J. Chapman

**Affiliations:** 1 Centre of Excellence for Autoimmunity in Cancer, School of Medicine, University of Nottingham, Nottingham, United Kingdom; 2 Oncimmune Ltd, Nottingham, United Kingdom; 3 Nottingham Digestive Diseases Biomedical Research Unit, Queen's Medical Centre, Nottingham, United Kingdom; 4 Institute of Clinical Chemistry and Clinical Pharmacology, University Hospital Bonn, Bonn, Germany; Taipei Medicine University, Taiwan

## Abstract

**Background:**

Individuals with liver disease, and especially those with Hepatitis B or C, are at an increased risk of developing hepatocellular carcinoma (HCC) which is the third most common cause of cancer-related death worldwide. Inadequate screening tests largely account for presentation of advanced tumours and high mortality rates. Early detection of HCC amongst high-risk groups is paramount in improving prognosis. This research aimed to further characterise the previously described humoral immune response raised to tumour-associated antigens (TAAs) in the serum of patients with HCC.

**Methods:**

Serum from 96 patients with confirmed HCC, 96 healthy controls matched for age and sex, 78 patients with confirmed liver cirrhosis and 91 patients with confirmed chronic liver disease were analysed for the presence of IgG autoantibodies raised to 41 recombinant TAAs/antigen fragments by ELISA.

**Results:**

Varying autoantibody specificities (97–100%) and sensitivities (0–10%) were observed to individual TAAs. A 21-antigen panel achieved a specificity of 92% and sensitivity of 45% for the detection of HCC. This same panel identified 21% of 169 high-risk controls as having elevated autoantibody levels. A reproducible panel of 10 antigens achieved a specificity of 91% and sensitivity of 41% in HCC. 15% of 152 high-risk controls gave positive results with this panel.

**Conclusions:**

This minimally invasive blood test has the potential to offer advantages over currently available tools for the identification of HCC amongst pre-disposed patients. Results are comparable to current gold standards in HCC (Ultrasonography) and to similar tests in other cancers (*Early*CDT-Lung).

## Introduction

Hepatocellular carcinoma (HCC) is the sixth most common cancer and the third most common cause of cancer-related death worldwide. Inadequate screening tests largely account for presentation of advanced tumours, poor prognosis and high mortality rates [1]. Between 1975 and 2009 in Great Britain, the age standardised incidence rates of liver cancer increased from 1.4 to 4.7 per 100,000 populations, reflecting what has become a global rise in HCC incidence. Approximately 3,960 new cases of liver cancer were diagnosed in the UK in 2009, leading to 3,800 liver cancer deaths in 2010 [2].

Worldwide, HCC incidence rates vary greatly and reflect the geographic distribution of risk factors (infection with hepatitis B or C viruses accounting for 85% of all HCC cases). HCC is most prevalent in the developing world; 82% of diagnosed HCC and HCC-related deaths occur in countries located in Southeast Asia and sub-Saharan Africa [3]. Despite its relatively lower prevalence, countries such as the USA and Japan are now also falling victim to the HCC burden [4].

For many years, surveillance of patients with pre-disposing liver conditions has comprised bi-annual serological screening tests (alpha-fetoprotein (AFP)) and/or imaging examinations (ultrasound), yet sensitivity for the detection of early stage HCC is universally considered sub-optimal. Measurement of AFP-L3 has also been investigated however a recent meta-analysis comparing AFP with AFP-L3 did not infer any significant improvement in cancer detection over AFP alone [5].

Recent investigations into the effect of applying AFP testing in addition to ultrasonography for surveillance of early HCC found that the marginal increase in sensitivity from 63% to 69% was not statistically significant. The use of AFP in surveillance of HCC is therefore not recommended by the most recent clinical practice guidelines [6]. Early detection of HCC amongst high-risk groups is paramount in improving prognosis, through enabling curative treatment options to be administered prior to manifestation of advanced and metastatic disease.

The presence of an elicited humoral immune response, in the form of IgG autoantibodies raised to Tumour-Associated Antigens (TAAs) in the sera of cancer patients, is well evidenced in the literature. Autoantibodies can be produced in response to mutated, over- or aberrantly expressed TAAs and may provide an *in vivo* amplification in patient sera of early carcinogenesis [7]. The presence of autoantibodies to TAAs has been described in several tumour types including breast [7-9)] ovarian [10], gastric [11] lung [12]-[15], colorectal [16], pancreatic [17] and oesophageal [18], and may be present years before clinical manifestation of the disease [19]–[23].

Techniques such as SEREX [24] T7 phage display [25], two-dimensional gel electrophoresis (2DE) and liquid chromatography-tandem mass spectrometry (LC-MS/MS) [26], amongst others, have been successfully employed for the detection of AAbs raised to TAAs in HCC and in patients with pre-disposing liver disease [27], [28]. However, previous studies have generally been performed using relatively small numbers of TAAs and with inappropriate control groups.

The aim of this study was to compare the performance of 41 TAAs/antigenic fragments in detecting a specific autoantibody response in the sera of patients with HCC using a high-throughput Enzyme Linked Immunosorbant Assay (ELISA). In contrast to many other published studies [27], [28], this study used control sera from age- and gender-matched individuals in order to show a true cancer versus ‘normal’ differentiation.

## Materials, Patients and Methods

This research was approved by the authors' institutional review boards and samples collected following approval by the University of Nottingham Research Ethics Committee (REC), Derbyshire REC and by the University of Munich Ethics Committee at the Medical Faculty of the Ludwig-Maximilians University, Munich). All samples were collected with written, informed consent at each of the respective collection centres. Sera were stored at −70°C prior to use.

### Selection of TAAs for Analysis

Using the literature as a source, antigens were selected for use in this study based on i) their association with HCC or liver disease and a previously uncharacterised autoantibody profile e.g. AFP, Gankyrin and GPC-3 or ii) proteins with a demonstrable immunogenicity in HCC [27], [28]; IMP1, p62, Koc, p53, c-myc, Cyclin B1, Survivin and p16, or other solid tumours, e.g. lung [21], [29]; SOX-2, CAGE, NY-ESO-1, GBU4-5, MAGE A-4, and HuD. Full-length recombinant proteins were produced where possible. In cases where PCR failed to amplify from template cDNA, primers were designed to allow the amplification of antigenic fragments of interest, e.g. the specific *thioesterase* domain of FASN was chosen for selective amplification due to its region-specific association with cancer [30].

### Serum Samples and Patient Details

#### HCC

57 HCC serum samples were collected (within 6 months of HCC diagnosis) at the Queen's Medical Centre, Nottingham, UK and 50 HCC serum samples were collected from the University Hospital Munich, Germany. A further 9 HCC serum samples were purchased from the Clinical Research Centre of Cape Cod. HCC diagnosis was confirmed either by BCLC staging classification [6] or as per Barcelona EASL Conference 2000 criteria [31].

#### Controls

169 samples from patients enrolled in the Trent study of patients infected with hepatitis C with either cirrhosis (n = 78) or chronic liver disease (n = 91) were used as high-risk controls. Healthy control samples were age- and gender- matched from a cohort of over 3,500 sera collected from healthy individuals in the East Midlands Area, with no evidence of liver disease.

### Tumour-Associated Antigen (TAA) Production

41 proteins, as well as a control antigen, BirA (which encodes a 14 kDa BirA recognition sequence) were produced as described below (for further details see supporting information, ). Where possible, cDNAs were sourced from sequence verified IMAGE clones (Source Bioscience). Alternatively, cDNA was synthesised from Huh7 cell line mRNA according to the manufacturer's instructions (QuantiTect Reverse Transcription Kit, QIAGEN). Full length antigens or antigenic fragments were either i) sub-cloned into pET21b-BirArs as previously described [32], [33] or ii) sub-cloned into a C-terminally tagged Ligation Independent Cloning (C-LIC) vector containing BirArs [34].

TAA-containing vectors were transformed into *E.coli* and cultured as either i) 30 ml cultures in deep well plates (n = 26) ii) as 200 ml shake-flask cultures (n = 8) or iii) as 5–15 L cultures (n = 8). Proteins were purified as previously described using IMAC His-Select filter plates (Sigma) [34] (n = 26) or His-trap FF-crude columns (GE) [25], [26] (n = 16). Fifteen promising antigens were also remade in 200 ml shake-flask cultures and purified using His-trap FF-crude columns, for analysis of antigen reproducibility.

### Autoantibody Detection

Autoantibodies were detected by ELISA according to previously described methods [34]. The BirA control was included to allow subtraction of any assay signal due to nonspecific binding.

In brief 41 TAAs/antigenic fragments (see supporting information), and 2 assay controls (BirA and buffer only) were adsorbed to ELISA plates in duplicate and at 2 concentrations of antigen (100 nM and 50 nM). These proteins were then incubated with serum samples from cancer, high-risk and healthy control cohorts (Table 1) diluted 1 in 110 in blocking buffer. The presence of an IgG response to the antigens was detected with horseradish peroxidase-conjugated rabbit anti-human IgG (Dako) and 3,3′,5,5′-tetramethylbenzidine, as previously described [29]. All assays were conducted on a semi-automated robotic system and cancer, high-risk and healthy control samples were interspersed. Incubations with anti-His monoclonal antibody (AbCam) and, where available antigen-specific monoclonal antibodies (Sigma, AbCam, Santa-Cruz), were carried out to validate antigen plate coating. SDS-PAGE analysis of TAA plate-coating solutions was also carried out to verify plate layouts and protein dilutions.

**Table 1:** Sample Cohort Information.

Sample Cohort	Total Number	Disease Aetiology	Mean Age	Sex
		HBV	HCV	ALD	Other	U/K		M	F
HCC	96	4	24	23	16	29	65	78	18
Healthy Controls	96	-	-	-	-	-	64	78	18
Cirrhotic	78	8	59	7	4	0	52	53	25
Chronic Liver Disease	91	34	56	1	0	0	43	59	32
Total number of samples	361	46	139	31	20	29	56	268	93

HBV  =  Hepatitis B Virus, HCV  =  Hepatitis C Virus, ALD  =  Alcoholic Liver Disease, Other  =  Primary Biliary Cirrhosis, Autoimmune Hepatitis, Haemochromatosis, 1 or more aetiologies, no underlying liver disease, U/K  =  Unknown.

### Autoantibody Data Analysis

Raw OD data was imported from the Tecan Infinite plate reader into Microsoft Excel for analysis using purpose-designed spreadsheets. The mean OD reading of the BirA control was subtracted from the mean OD readings of antigen-coated wells. Intra-assay reproducibility was determined by calculating the Coefficient of Variation (CV) for each dilution.

Optimised antigen cut-offs (for maximal cancer: normal differentiation), as well as a standard cut-off that corresponded to a value greater than the mean plus 4 Standard Deviations (SD) of the healthy control cohort, were applied to each antigen. Statistical analysis was performed using Microsoft Excel, IBM SPSS Statistics 21 or GraphPad Prism 6. The 2-sample Kolmogorov-Smirnov test was used for the analysis of two separate distributions and the 1-sample Kolmogorov-Smirnov ‘goodness of fit’ test employed to analyse the normality of the data [35].

Significance analysis was subsequently performed using the Mann-Whitney U and Pearson Chi-Square tests on non-parametric data. Overall data sensitivity and specificity were estimated to determine the usefulness of autoantibody detection, in terms of diagnostic potential.

## Results

Sample cohort demographics and details of the underlying disease aetiology are included in Table 1. No difference was observed in autoantibody signal between UK and German HCC sample cohorts.

### Analysis of Individual Antigens

**Figure 1:**
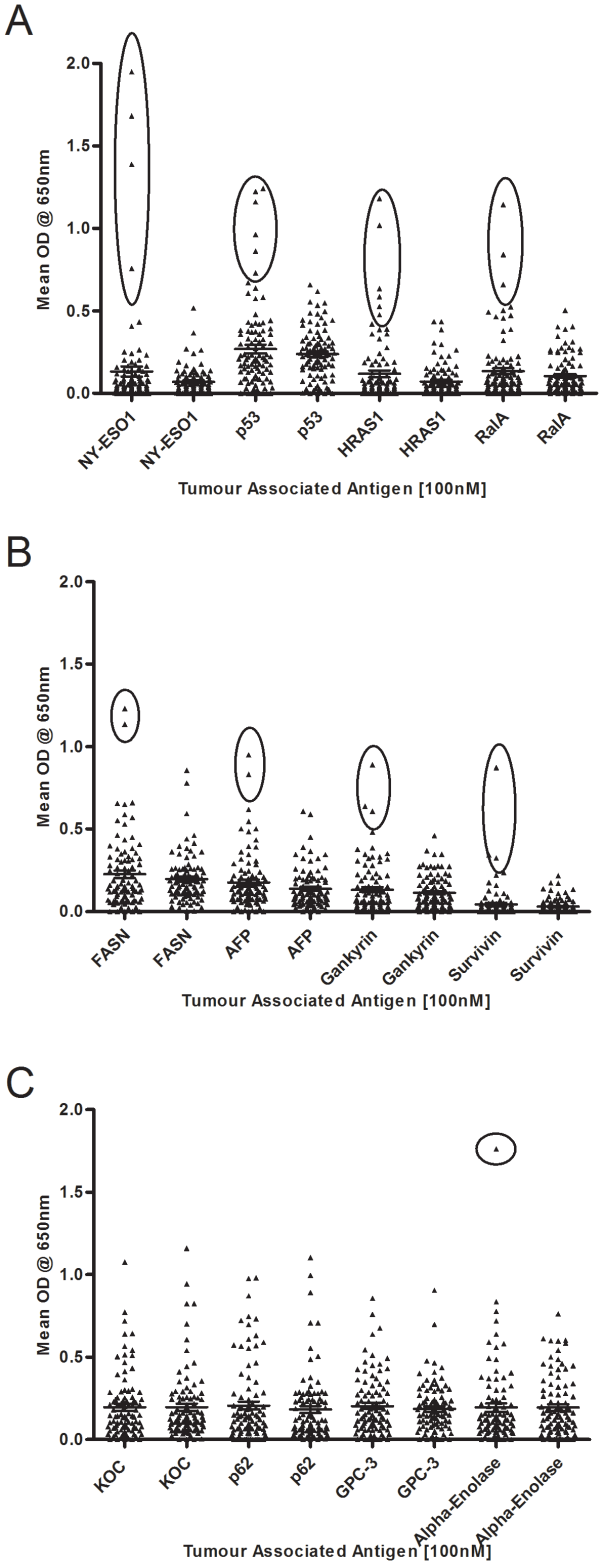
Dot plots of the mean OD autoantibody signal received from each serum sample against 9 TAAs. NY-ESO-1, p53, H-RAS-1 and RalA (A), FASN, AFP, Gankyrin and Survivin (B) and KOC, p62, GPC-3 and Alpha-enolase (C). Data for each antigen is shown as cancer then matched control. Error bars signify the mean with 95% confidence interval. Circled data points identify positive HCC responses.

Antigens were initially grouped according to their K-S scores (data not shown), analysis of dot blots and their ability to differentiate between cancer and healthy control (‘normal’) cohorts. Details of the antigens analysed, and individual sensitivities for 21 of the antigens are shown in table 2 (study 1). The other 20 antigens demonstrated no cancer: normal differentiation and are shown as a group of rejected antigens. Examples of dot blots for some of the antigens are exemplified in Fig. 1. Some TAAs such as NY-ESO-1, p53, HRAS1 and RalA showed good differentiation between HCC and healthy control sera, as shown by the circled cloud of positive samples in the HCC cohort. Other TAAs such as FASN, AFP, Gankyrin and Survivin showed much less differentiation whilst KOC, p62, GPC-3 and Alpha-enolase showed extremely poor differentiating ability between cohorts.

**Table 2:** Comparison of individual antigen specificities and sensitivities.

TAA	STUDY 1	STUDY 2
	Specificity (%)	Sensitivity (%)	High Risk Positivity	Specificity (%)	Sensitivity (%)	High Risk Positivity
			(%) n = 169)			(%) (n = 152)
AFP	97	8	3	96	8	4
Cyclin B1	97	10	5	97	8	5
Gankyrin	99	5	4	99	5	2
p53	100	7	4	98	7	3
NY-ESO-1	100	5	2	100	5	1
RalA	99	10	4	99	4	3
CK8	100	3	1	100	5	3
GRP78	98	6	3	98	4	4
HDGF	98	6	3	97	6	1
DKK1	99	0	0	99	3	0
H-RAS-1	100	7	3	100	1	1
p16	99	6	4	99	1	2
WT1 (n terminal)	99	6	2	99	1	1
HCC1	100	4	1	100	0	0
Sui1	98	8	3	98	4	4
l-myc2	98	4	2	98	3	4
GPC-3	98	10	3	98	0	1
Beta-Catenin2	97	5	1	97	1	6
Beta-HCG	99	8	0	98	2	2
Calreticulin	99	3	1	99	1	0
FASN	100	2	0	100	2	1
21 Antigen Panel (above)	92	45	21	88	43	n/a
Rejected Antigen Panel (n = 20)	80	23	n/a	n/a	n/a	n/a

Data shown for 96 cancers (% sensitivity), 96 matched normal sera (% specificity) and at risk sera (% positivity). Study 1: Data from initial screen of 41 antigens. Study 2: Data from the 21 lead antigens. Individual cut-offs for each antigen were applied to maximise cancer: normal differentiation.

TAA – tumor-associated antigens. n/a – not assessed.

Rejected antigens  =  Vitronectin; Survivin; KOC; p62; α-enolase; c-myc; GBU4-5, β-Catenin 1; CAGE; HuD; H-RAS-2; PRDX6; IMP1; K-RAS; MAGE-A4; MAGE-C2; SOX2; SSX1; VEGF-C; WT1-c-terminal.

**Figure 2:**
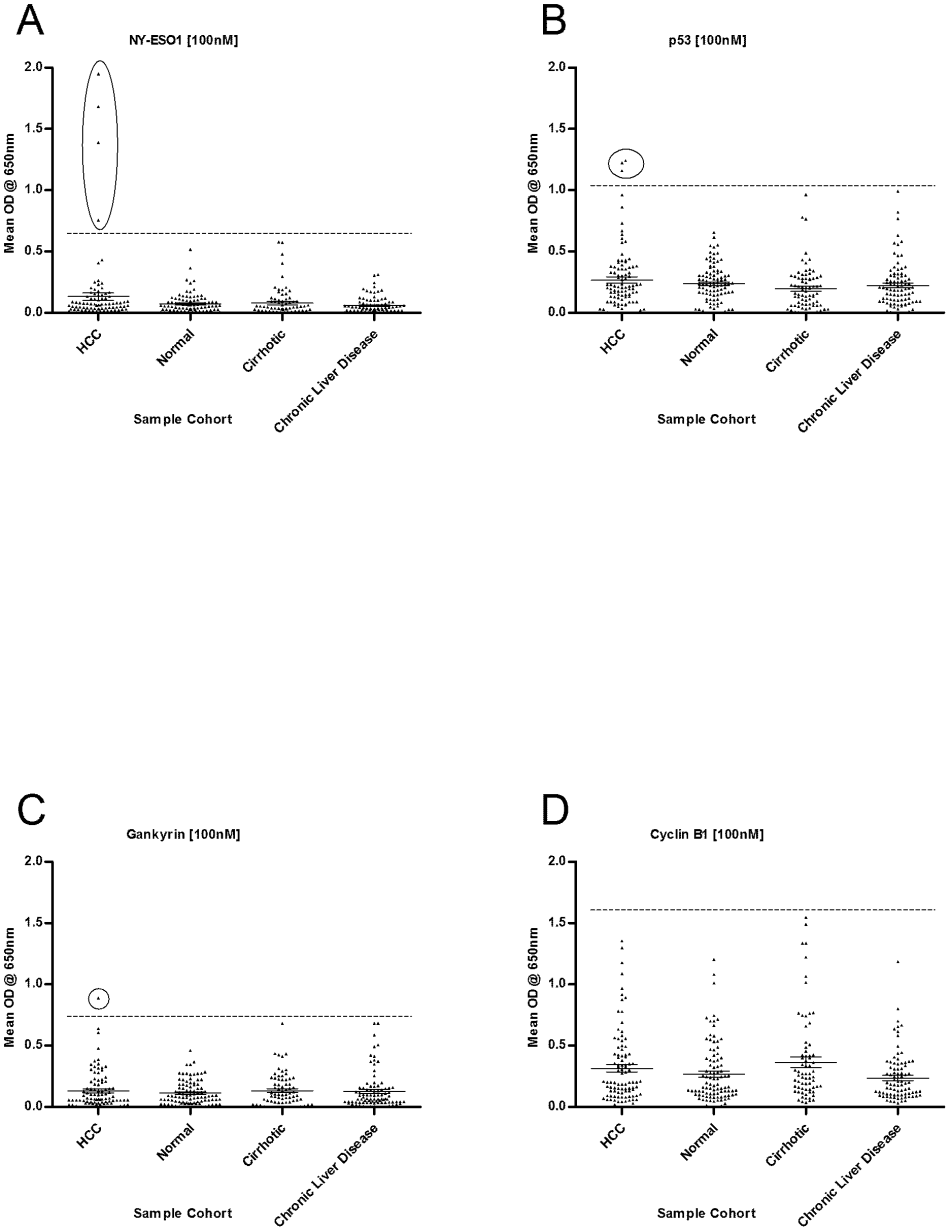
Dot plots of the mean OD autoantibody signal received from each serum sample from all four cohorts, against 4 TAAs. (NY-ESO-1 (A), p53 (B), Gankyrin (C) and Cyclin-B1 (D). Error bars signify the mean with 95% confidence interval. Circled data points identify positive HCC responses. Horizontal lines above data sets indicate the OD value of mean plus 2SD of the normal control cohort.

Antigens were also grouped according to their ability to differentiate between cancer and high-risk sample cohorts. Dot blots for some of the antigens are exemplified in Fig. 2; some antigens such as NY-ESO-1 maintained the promising differentiation shown previously between cancer and healthy control cohorts. Other TAAs such as p53 and Gankyrin showed reduced cohort differentiation and Cyclin B1 showed no cohort differentiation.

### Antigen Panel Selection

Further analysis of the 41 antigens (Table 2), using a standardised cut-off for each antigen of an OD value equal to or greater than the mean plus 4SD of the healthy control cohort, identified 5 discrete TAA panels of between 1 and 21 antigens (Table 3). Testing was based on 96 HCC and 96 matched healthy control samples. The percentage of high-risk samples with elevated autoantibody levels was also investigated for each antigen panel. Sensitivity for cancer detection increased from 7% to 41% with a 12 antigen panel, and to 45% for a 21 antigen panel, whilst specificity was maintained at >90%. In all cases more high-risk individuals than normal matched controls were identified.

**Table 3:** Comparisons of specificity and sensitivity in 5 different TAA panels.

Number of TAAs in panel	Specificity/Sensitivity (%)	High-risk Positivity (%) (n = 169)
**1**	100/7	3
**4**	96/23	9
**8**	96/30	14
**12**	92/41	18
**21**	92/45	21

Panel composition was as follows:1 =  H-RAS-1;4 =  H-RAS-1 + p16 + Gankyrin + NY-ESO-1;8 =  Panel 4 + Sui1, p53, RalA, CyclinB1;12 =  Panel 8 + HCC1, WT1-n-term, CK8, AFP;21 =  Panel 12 + β-Catenin2, β-HCG, HDGF, Calreticulin, GRP78, FASN, GPC-3, DKK1, l-myc-2.

### TAA panels and association with HCC stage and aetiology

45% of HCC patients tested were identified as positive to one or more TAAs. Sub-analysis of autoantibody positivity rates in tumours of different aetiologies was not possible, owing to lack of aetiological data relating to many of the HCC samples. Similarly, we were not able to discern whether reactivity with any particular TAA was related to underlying HCC aetiology.

Clinical information on tumour stage was limited. 16 patients were known to have early stage disease (BCLC Stage A) at the time of venous puncture. Of these, 11 were initially identified as positive (positivity to 1 or more TAAs from 41 TAAs tested), of which 8 were autoantibody positive in the 21 TAA panel.

Sera prior to HCC diagnosis were available from 11 patients. Seven of these had raised autoantibody levels to at least 1 TAA (from a panel of 12) up to 5 years before the cancer had been clinically diagnosed.

### Normality and Significance testing

Normality testing classified all data as non-parametric (data not shown). Mann-Whitney U significance data confirmed that l-myc-2 (p = 0.03) was the only antigen to significantly differentiate between cancer and healthy serum cohorts (HCC > healthy controls), and AFP-C (p = 0.04), NY-ESO-1 (p = 0.04) and DKK1 (p = <0.01) were the only antigens to significantly differentiate between cancer and high-risk serum cohorts (HCC > high-risk controls). The 21 TAA panel achieved significant differentiation between all sample cohorts (p = <0.01).

### Panel Reproducibility

The 21 best performing TAAs were selected for closer analysis. Five of these had already been produced in large scale and 16, initially derived from small-scale production, were re-purified on a larger scale. Following larger scale purification varying autoantibody specificities (96%–100%) and sensitivities (0%–8%) were again detected to each of 21 TAAs in the sera of patients with HCC and patients with liver disease compared to serum from healthy volunteers, confirming the initial results from this study (table 2, study 2). Following optimisation of the cut off for the detection of HCC samples (when compared to matched healthy controls), the 21 antigens were able to identify 43% of the HCC samples with a slightly reduced specificity of 88%.

To increase the utility of a panel of antigens, and remove redundant antigens (eg those that were found to be non-reproducible following high volume purification, or those that did not identify unique cancers) sub-panels were again investigated. Seven of the antigens were found to still differentiate between cancer, normal and high risk sera (AFP, Cyclin B1, Gankyrin, p53, NY-ESO-1, RalA, CK8) whilst other promising antigens H-RAS-1, p16, WT1, HCC1 and Sui1 were no longer found to be additive.

A reproducible sub-panel consisting of these 7 TAAs plus 3 others (GRP78, HDGF and DKK1) gave a specificity and sensitivity for identification of HCC of 91%/41% when compared to matched healthy control sera. 15% of ‘at risk’ individuals were also found to give positive results with this panel.

## Discussion

Serum autoantibody detection has been proposed as an effective aid to the early identification of HCC in patients considered at increased risk of cancer development [27], [28], [36]. A reproducible panel of 10 TAAs, was found to carry a diagnostic specificity of 91% and sensitivity of 41%. Such a specificity and sensitivity is a comparable diagnostic accuracy to that reported as being of clinical utility in lung cancer [14], [29] and with further optimisation, has the potential to improve on the diagnostic accuracy for early stage disease, offered by gold standards in HCC (AFP/US imaging) [6].

This study improves on previous studies reporting on autoantibodies in HCC, as there are a number of limitations in many of the published studies including numbers of TAAs tested per study - e.g. Zhang et al and Chen *et al.* used only 10 TAAs [27], [28]; the inappropriate use of un-matched ‘normal’ control groups, and low numbers of high-risk samples potentially introducing various biases in age, gender and socioeconomic class into the control cohort [27], [28]. In addition few studies report whether or not their findings were reproducible when run on a different day with a second batch of the same proteins. This study aimed to address these limitations by firstly investigating the autoantibody response to an initial panel of 41 TAAs/antigenic fragments; secondly, through screening a more robust sample set; 96 HCC, 96 age- and gender-matched healthy control sera, 91 chronic hepatitis and 71 liver cirrhosis serum samples (where possible, healthy control sera were also matched to HCC samples according to smoking history) and thirdly we produced different batches of the same protein and re-ran the samples to show reproducibility of results.

The 21 best performing TAAs selected for closer analysis included TAAs previously identified as promising leads in HCC such as IMP-1, KOC, p53 and c-myc [27], Sui1 and RalA [28], Calreticulin [37], and HCC1 [38] together with novel proteins such as Gankyrin and FASN, and well-known liver biomarkers such as AFP, GPC-3 and GRP78. Testing a large number of TAAs i) enabled the identification of several new leads including HRAS1, Gankyrin, and CK8, ii) confirmed some leads previously published including RalA, Sui1 and p53 [28] and in other cases, iii) contradicted previous reports on promising antigen performance, such as KOC, p62 and c-myc [27]. Reasons for discrepancies between published studies will be multi-factorial, including sample size, and selection of more or less appropriate control groups as well as antigen production methodologies. Differences in sample set demographics may also be relevant, reflecting differing HCC aetiologies in different studies.

The importance of optimal antigen production was highlighted in this study given that not all antigens that initially displayed a good differentiating ability between cancer and healthy controls, maintained their ability to do so on re-purification. These results demonstrate the need for optimal antigen production and validation before commercialisation of such tests. Alternative methods for production of these antigens (in terms of expression and purification) may, in future, enable them to be included in a test for HCC.

The promise held by panel autoantibody detection has previously been evidenced in HCC as well as other cancers such as lung [12]–[14], [29]. Our results confirm that no single antigen alone can identify large numbers of positive samples. We have shown that increasing the TAA panel to include 21 as opposed to 4 antigens resulted in a doubling of sensitivity from 23% to 45% whilst specificity was only reduced from 96% to 92%. We had access to a well characterised cohort of sera from healthy volunteers and at risk individuals, thereby enabling crucial age- and gender- matching of HCC samples, and analysis of autoantibody patterns in important at-risk groups. We were also able to use a technically and clinically validated assay platform technology thereby ensuring autoantibody assays were conducted in a highly reproducible manner [32], [33]. We note that Zhang *et al*, and Chen *et al*. reported sensitivities of 67% to a panel of 10 antigens [27], [28], however, their failure to use appropriately age- and sex-matched controls leaves a significant clinically relevant question on their assay performance unanswered.

One limitation of our study is the lack of aetiological information for many of our HCC sera, which precludes analysis of whether TAA panels could be tailored to detect specific aetiological sub-types of HCC. However, even if the data for all 96 cases was available, the numbers would have been too small for each of the main aetiological causes (ie HBV, HCV and alcohol) to infer panel preferences. This however remains an attractive area for further research.

Of particular interest is the evidence that autoantibody responses can be detected in patients at an early stage of disease (BCLC stage A [6]) and in some cases, up to 5 years prior to clinical diagnosis. This is in keeping with previous reports where autoantibodies to TAAs have been reported between 0.5–4 years before symptomatic presentation in lung, breast and colon cancer [20]–[23] and up to 5 years before detection of lung cancer in a CT screening study [19].

The reproducible panel of 10 TAAs, included novel HCC antigens such as Gankyrin and CK8, achieved the specificity of 91% and sensitivity of 41%, even upon partial scale-up of antigen and despite the fact that 3 of the originally identified antigens were no longer found to be additive to the panel, illustrating that optimisation of protein production prior to commercial launch of a test, is paramount.

Autoantibodies to 10 antigens were also evident at raised levels in 15% of at risk individuals. One possible reason for the positivity amongst the high risk group is the presence of a developing but as yet undiagnosed HCC. If longitudinal studies are carried out in the future, and this group do indeed go on to develop HCC and the remaining do not, it is possible to speculate that the test could be detecting an immune response to a few early HCC cells present in the liver of such patients. Clearly future studies with appropriate follow-up will be needed to address this hypothesis, however this may prove to be a significant group to follow, as the five-year cumulative risk for HCC in patients with HCV-related cirrhosis can be as high as 17% in Europe and the US, and 30% in Japan [39].

A simple blood test, such as described here, would, once optimised and validated, have the potential to offer an aid to the clinician in assessing individuals at increased risk of developing the disease. The ultimate aim of which would be the reduction of lives lost to this malignancy through its detection at an early stage.

## Supporting Information

Table S1 **TAA Production.** (A  =  30 ml culture volumes and HIS-Select filter plate purification; B and C  =  200 ml and >5 L culture volumes respectively with HIS-Trap FF-crude Fast Protein Liquid Chromatography purification). * Denotes molecular weight including BirA tag.
(DOCX)
