# Supplementary material for: Serum Autoantibody Measurement for the Detection of Hepatocellular Carcinoma
Source: PLoS One. 2014 Aug 5;9(8):e103867. doi: 10.1371/journal.pone.0103867 (PMC4122394; doi:10.1371/journal.pone.0103867)
Supplement: Table S1 — TAA Production. (A = 30 ml culture volumes and HIS-Select filter plate purification; B and C = 200 ml and >5 L culture volumes respectively with HIS-Trap FF-crude Fast Protein Liquid Chromatography purification). * Denotes molecular weight including BirA tag. (DOCX) [file pone.0103867.s001.docx]

**Table S1.**  **TAA Production**

(A = 30 ml culture volumes and HIS-Select® filter plate purification; B and C = 200ml and >5L culture volumes respectively with HIS-Trap FF-crude Fast Protein Liquid Chromatography purification). * Denotes molecular weight including BirA tag.

|  | Antigen | Full Length (FL) / Fragment (F) | Vector | Size * (kDa) | Production Method |
| --- | --- | --- | --- | --- | --- |
| Selected Panel of 21 TAAs | HRas1 | F | C-LIC | 32 | A &B |
|  | p16 | F | C-LIC | 26 | A &B |
|  | Sui-1 | FL | C-LIC | 28 | A &B |
|  | WT1-n term | F | pET21b-BirA | 49 | B |
|  | p53 | FL | C-LIC | 59 | C |
|  | Ral A | FL | C-LIC | 38 | A & B |
|  | Gankyrin | FL | C-LIC | 39 | A & B |
|  | NY-ESO-1 | FL | pET21b-BirA | 34 | C |
|  | GRP78 | FL | C-LIC | 87 | A & B |
|  | CK8 | FL | C-LIC | 69 | B |
|  | AFP | FL | C-LIC | 84 | A & B |
|  | Cyclin B1 | FL | C-LIC | 63 | A & B |
|  | HCC1 | FL | C-LIC | 74 | A & B |
|  | HDGF | FL | C-LIC | 42 | A & B |
|  | Calreticulin | FL | C-LIC | 63 | A & B |
|  | GPC3 | FL | C-LIC | 78 | A & B |
|  | Beta-HCG | FL | C-LIC | 33 | A & B |
|  | L-myc-2 | FL | C-LIC | 37 | B |
|  | FASN | F | C-LIC | 49 | A & B |
|  | Beta-Catenin 2 | F | C-LIC | 60 | A & B |
|  | DKK1 | FL | pET21b-BirA | 44 | B |
| Rejected Panel of 20 TAAs | Vitronectin (VTN) | F | C-LIC | 24 | A |
|  | Survivin | FL | pET21b-BirA | 31 | A |
|  | KOC | FL | C-LIC | 79 | A |
|  | p62 | FL | C-LIC | 81 | A |
|  | Alpha-enolase | FL | pET21b-BirA | 63 | B |
|  | C-myc | FL | pET21b-BirA | 64 | B |
|  | GBU 4-5 | FL | pET21b-BirA | 58 | C |
|  | Beta-Catenin 1 | F | C-LIC | 56 | A |
|  | CAGE | FL | pET21b-BirA | 87 | C |
|  | HuD | FL | pET21b-BirA | 55 | C |
|  | HRas 2 | F | C-LIC | 19 | A |
|  | PRDX6 | FL | C-LIC | 40 | A |
|  | IMP1 | FL | C-LIC | 78 | A |
|  | KRAS | FL | C-LIC | 36 | A |
|  | MAGE A4 | FL | pET21b-BirA | 51 | C |
|  | MAGE-C2 | FL | pET21b-BirA | 56 | A |
|  | SOX2 | FL | pET21b-BirA | 50 | C |
|  | SSX1 | FL | C-LIC | 37 | A |
|  | VEGFC-M-C-BirA | F | pET21b-BirA | 28 | B |
|  | WT1-c-term-BirA | F | pET21b-BirA | 50 | B |
| Control | Bir A |  | pET21b | 14 | C |
